# Methanol Decomposition on TiO_2_ Under Ambient Conditions: Cooperative Proton Transfer at Hydrated Interfaces

**DOI:** 10.1002/anie.3448039

**Published:** 2026-09-07

**Authors:** Milad Salarinasab, Julian Andreas Hochhaus, Jing Wang, Aimeric Ouvrard, Anas Akhtar, Michele Capra, Kristina Tschulik, Carsten Westphal, Mirko Cinchetti, Bo Wen, Li‐Min Liu, Zhe Wang, Ahmed Ghalgaoui

**Affiliations:** ^1^ Department of Physics TU Dortmund University Dortmund Germany; ^2^ School of Physics and Electronics Henan University Kaifeng China; ^3^ Institute des Sciences Moléculaires d'Orsay (ISMO) Paris Saclay University Orsay France; ^4^ Chair of Analytical Chemistry‐ Electrochemistry and Nanoscale Materials Ruhr‐Universität Bochum Bochum Germany; ^5^ School of Physics Beihang University Beijing China

**Keywords:** interfacial hydration, methanol oxidation, photooxidation, solid–liquid interface, TiO_2_ photocatalysis

## Abstract

Methanol decomposition is a benchmark reaction for understanding surface‐mediated redox chemistry and photocatalytic processes on oxide materials. While its mechanism on TiO_2_ has been extensively studied under ultra‐high‐vacuum (UHV) conditions, the reaction pathway under ambient, liquid‐phase environments remains largely unexplored. Here, we investigate the photocatalytic decomposition of methanol on rutile TiO_2_(110) following direct adsorption from liquid solution by combining surface‐sensitive sum frequency generation (SFG) spectroscopy with density functional theory (DFT) calculations. SFG measurements identify methoxy as the dominant surface species under these conditions. DFT calculations reveal that cooperative hydrogen‐bonding interactions stabilize both the transition state and the dissociated configuration, leading to a systematic reduction in activation barriers. Concurrently, localized excess charge facilitates proton transfer through dynamic redistribution along the reaction coordinate, thereby lowering the barrier for O─H bond cleavage and stabilizing reaction intermediates. Under resonant bandgap excitation, methoxy undergoes structural reorientation followed by oxidation to form a stable surface hydroxymethyl species. DFT calculations further demonstrate that interfacial water and hydroxyl groups stabilize localized charge carriers, reduce activation barriers, and promote proton‐coupled electron transfer. These findings provide molecular‐level insight into the role of interfacial hydration in governing photocatalytic reaction pathways at oxide–liquid interfaces.

## Graphical abstract

Under ambient conditions, the TiO_2_ surface becomes hydrated by adsorbed hydroxyl groups (OH) and water molecules. This surface hydration promotes methanol decomposition, rendering the first deprotonation step nearly barrierless compared with ideal UHV conditions. Under UV illumination, surface hydration further facilitates methoxy oxidation through stabilization of localized charge carriers and cooperative proton transfer. Thus, ambient conditions enhance photocatalytic alcohol oxidation.

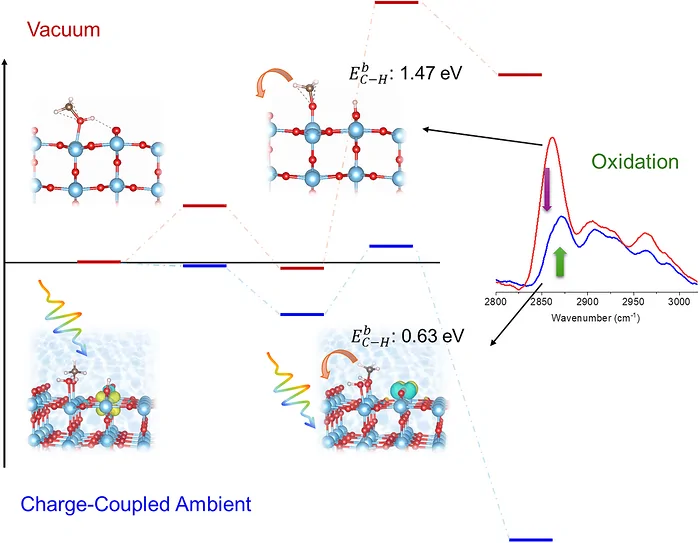

## 1 Introduction

Methanol has emerged as a key molecular platform for energy conversion, functioning both as a liquid fuel, and as a compact hydrogen carrier owing to its high hydrogen density and ease of catalytic conversion [1, 2, 3, 4, 5, 6]. Beyond its technological relevance, methanol decomposition represents a prototypical reaction for elucidating surface‐mediated redox chemistry and photocatalytic processes on oxide materials. Among these systems, titanium dioxide (TiO_2_) stands out as an earth‐abundant, chemically robust, and environmentally benign semiconductor photocatalyst that has attracted sustained attention since the discovery of its photocatalytic activity [7, 8, 9, 10, 11, 12]. Its structural simplicity, exceptional stability, and well‐established performance make TiO_2_ an ideal model system for fundamental investigations of surface reactivity [13, 14, 15].

The adsorption and decomposition of methanol on well‐defined TiO_2_ surfaces, including both rutile and anatase phases, have been extensively investigated under UHV conditions [16, 17, 18, 19, 20, 21]. These studies show that methanol adsorbs dissociatively to form surface‐bound methoxy species via proton transfer to bridging oxygen atoms. DFT further indicates that initial O─H bond cleavage proceeds with a relatively low activation barrier of ∼0.32 eV, whereas subsequent C─H activation is considerably less favorable, requiring ∼1.47 eV [22, 23, 24] and thus constituting the kinetic bottleneck for further decomposition.

Despite this detailed mechanistic picture, the behavior of methanol on TiO_2_ surfaces under ambient and liquid‐phase conditions remains comparatively underexplored. At the solid–liquid interface, solvent molecules and dynamic hydrogen‐bonding networks can substantially alter adsorption energetics, proton‐transfer pathways, and interfacial charge distributions [25, 26]. In addition, exposure to humidity can heal surface defects [27, 28, 29] and modify charge‐carrier dynamics by providing a stabilizing environment that prolongs electron and hole lifetimes, thereby enhancing photocatalytic activity [30, 31, 32, 33]. Under such conditions, the surface is expected to undergo extensive hydroxylation via dissociative adsorption of water and oxygen, giving rise to hydrated, dynamically evolving interfacial structures [19, 34, 35]. This hydration can further promote reactivity by facilitating proton‐transfer processes [32]. Recent theoretical studies further indicate that co‐adsorption of H_2_O and CH_3_OH promotes methanol dissociation on rutile while enhancing water dissociation on anatase [36]. Moreover, oxygen adsorption not only heals surface defects but also alters methanol photooxidation pathways, for example, by enabling methyl formate formation [37]. Collectively, these findings suggest that surface chemistry under ambient conditions can access reaction pathways and reactivity patterns that differ fundamentally from those observed under UHV conditions.

Under photocatalytic conditions, the coexistence of multiple co‐adsorbed species with distinct reactivities toward photogenerated charge carriers introduces an additional level of complexity. Methoxy species, for instance, exhibit markedly higher photoactivity than molecularly adsorbed methanol, consistent with their more efficient coupling to photogenerated holes [20]. Methanol photocatalysis is further modulated by surface hydroxyl groups, co‐adsorbed oxygen species, and surface coverage effects [32]. Notably, methanol displays negligible reactivity even under intense UV irradiation; photoactivation is observed only at high surface coverage or in the presence of OH^−^ groups or O_2_. These species promote proton‐shuttling processes and stabilize reactive intermediates, thereby enabling oxidation pathways that are otherwise inaccessible [32].

A further level of complexity arises from the interplay between site‐specific adsorption geometries and photogenerated charge carriers. Methanol dissociation predominantly yields methoxy species bound to fivefold‐coordinated Ti (Ti_5c_) sites; however, under illumination, subsequent reactivity is modulated by structural rearrangements and surface diffusion processes that are strongly influenced by hydration and hydrogen‐bonding interactions. Photoexcited holes promote the activation of these surface‐bound intermediates, while the resulting reaction pathways are strongly influenced by the local coordination environment and electronic structure of the oxide surface.

Concurrently, co‐adsorbed water and hydroxyl species may actively participate by facilitating proton transfer, stabilizing charged intermediates, and opening alternative oxidation pathways not accessible on dry surfaces. A molecular‐level understanding of how photoexcitation, site‐specific adsorption, and surface hydration collectively govern methanol oxidation is therefore essential for establishing realistic reaction mechanisms at oxide–liquid interfaces.

Building on the established UHV paradigm of methanol activation on TiO_2_(110) and the growing recognition that surface chemistry is fundamentally altered under realistic environments, we directly interrogate photocatalytic methanol decomposition under liquid‐phase conditions that closely mimic operational catalysis. We combine surface‐specific SFG spectroscopy with DFT calculations to achieve, for the first time, a molecularly resolved steady‐state description of interfacial methanol oxidation under continuous bandgap illumination. This approach enables direct identification and tracking of reactive intermediates at the buried solid–liquid interface under true photocatalytic operating conditions, thereby overcoming the long‐standing disconnect between UHV‐derived mechanisms and ambient photocatalysis. Our results demonstrate that surface hydroxylation, solvent‐mediated restructuring, and photogenerated charge carriers are not merely perturbations to an idealized surface reaction but instead collectively define the operative reaction landscape under illumination. This work establishes a unified molecular framework for photocatalytic alcohol oxidation at oxide–liquid interfaces, in which structure, solvation, and electronic excitation are intrinsically coupled under operating conditions.

## 2 Results and Discussion

### 2.1 Methanol Adsorption on Hydrated TiO_2_: Steady‐State Surface Characterization and Adsorbate Identification

Rutile TiO_2_(110) single crystals were prepared under UHV conditions by repeated cycles of Ar^+^ sputtering and annealing at 900 K, yielding a clean and well‐ordered surface. Surface structure under UHV was verified by low‐energy electron diffraction (LEED). Following exposure to ambient conditions, surface morphology was characterized by atomic force microscopy (AFM), revealing large terraces up to ∼200 nm in width. Details of the sample preparation, as well as LEED and AFM characterization, are provided in (Figures S2–S4). Upon exposure to air, the surface is expected to be terminated by a mixture of hydroxyl groups formed through dissociative adsorption of water (O_br_H and O_t_H), together with molecularly adsorbed water and trace contaminants. Ambient exposure of TiO_2_ is also known to result in the adsorption of adventitious hydrocarbons and trace atmospheric carboxylic acids, which can form strongly bound carboxylate species at the surface [38]. The presence of water was verified using XPS after exposure to air, revealing a shoulder at 532.2 eV in the O1s region, indicative of hydroxylated and molecularly adsorbed water species. Notably, this OH/H_2_O‐related feature remains observable after methanol immersion, confirming the coexistence of methanol‐derived species with surface hydroxyls and/or adsorbed water (Figure S5). SFG measurements in the C─H stretching region revealed features between 2830 and 2960 cm^−^^1^ arising from adventitious hydrocarbon species and potentially adsorbed organic acids containing alkyl groups. These species do not significantly affect the spectral assignments because their intensities are low relative to those of surface‐derived methoxy species directly formed upon adsorption from solution (Figure S6). For the methanol adsorption experiments, samples were immersed overnight in high‐purity methanol at room temperature to promote the formation of surface methoxy species prior to spectroscopic characterization.

We next examine methanol adsorption using surface‐sensitive SFG spectroscopy. Figure 1 shows the SFG spectra acquired during methanol decomposition on rutile TiO_2_(110). Spectral deconvolution reveals a dominant band at 2839 cm^−^^1^, assigned to the symmetric C─H stretching mode of methoxy species bound to fivefold‐coordinated titanium sites (CH_3_O–Ti_5_c).

**FIGURE 1:**
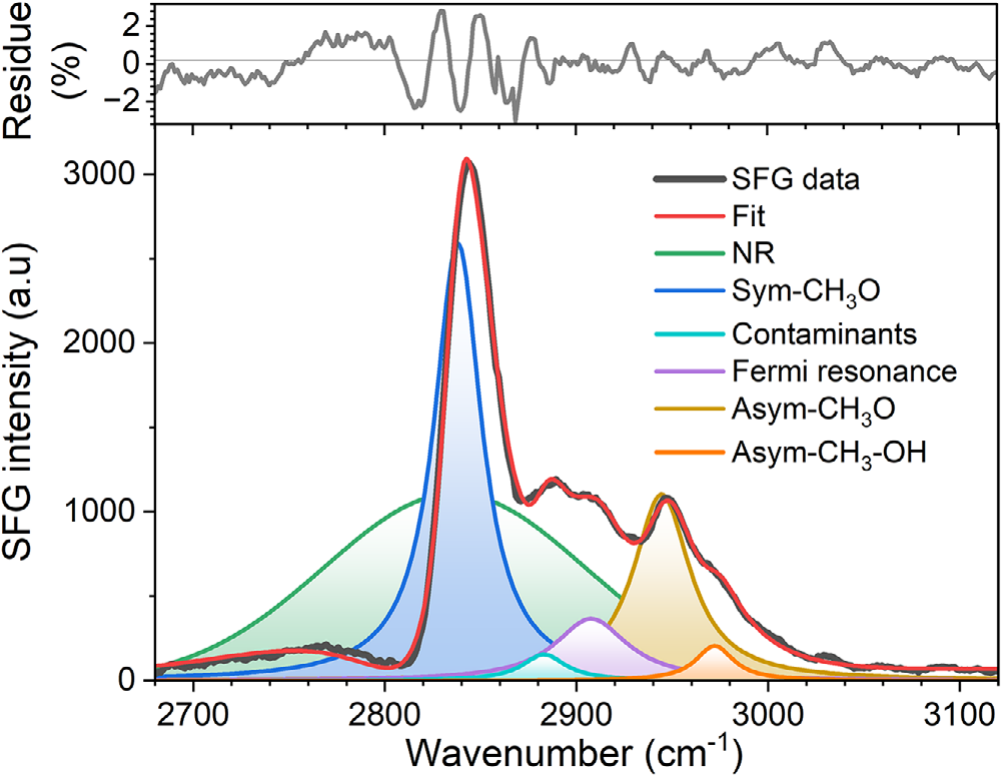
SFG spectrum of methanol adsorbed on rutile TiO_2_(110) from liquid solution. The black trace shows the experimental data, the red trace the spectral fit, and the green trace the nonresonant (NR) contribution. Spectral deconvolution reveals that the response is dominated by methoxy species bound to fivefold‐coordinated Ti sites (CH_3_O─Ti_5_c), with a minor contribution from molecularly adsorbed methanol.

Additional features in the 2860–2930 cm^−^^1^ region arise from Fermi resonance modes of the methyl group, overlapping with contributions from adventitious hydrocarbons. Bands at 2940 and 2970 cm^−^^1^ are assigned to antisymmetric C─H stretching vibrations of methoxy and molecular methanol, respectively. A band attributed to methyl stretching vibrations of molecularly adsorbed methanol, is expected at ∼2851 cm^−1^, however, it overlaps with the dominant methoxy band and was not included in the fit. The coexistence of methoxy and intact methanol is consistent with prior theoretical and experimental studies, which show that methanol adsorption on TiO_2_(110) proceeds predominantly via dissociation to methoxy, while a minor fraction of molecular methanol remains stable under ambient or near‐ambient conditions. In particular, ab initio thermodynamics and kinetic Monte Carlo simulations predict a stable partially dissociated adsorption phase in which methanol and methoxy coexist at intermediate coverages and temperatures (∼150–300 K) [39], whereas recent experimental studies indicate that the surface at room temperature is dominated by methoxy with a small but detectable contribution from molecular methanol [40]. This assignment is supported by the observation of additional CH_3_ antisymmetric stretching features attributable to molecular methanol, distinct from those associated with methoxy. The observed vibrational frequencies of the methoxy species are blueshifted by ∼6 cm^−^^1^ relative to UHV studies of methanol on TiO_2_(110) [41, 42], reflecting differences in the interfacial environment. In addition to hydrogen‐bonding interactions with co‐adsorbed hydroxyl groups and interfacial water, lateral interactions between neighboring methanol and methoxy species at higher surface coverage are expected to stabilize partially dissociated, hydrogen‐bonded configurations and contribute to the observed frequency shifts. Details of the fitting procedures are provided in the Supporting Information.

Methanol deposition from solution strongly favors the formation of surface methoxy species, as evidenced by the predominance of methoxy‐related vibrational signatures, in agreement with previous reports under near‐ambient conditions [39, 40]. This behavior underscores the stabilizing role of the hydrated interface, where co‐adsorbed water molecules and hydroxyl groups promote methanol deprotonation through cooperative hydrogen‐bonding interactions and surface acid–base chemistry. To elucidate the microscopic origin of this stabilization and clarify subsequent reaction pathways, we employ DFT) calculations to investigate methanol decomposition on TiO_2_(110) in the presence of water, hydroxyl species, and photogenerated charge carriers.

### 2.2 DFT Modeling of Methanol Adsorption on R‐TiO_2_(110) Under Ambient Conditions

To assess the influence of the local environment on methanol decomposition, DFT calculations were performed (computational details are provided in the Supporting Information). The TiO_2_ substrate was modeled as an unreconstructed rutile TiO_2_(110) surface using a 4 × 2 slab comprising four trilayers, separated by a vacuum region of ∼15 Å to minimize spurious interactions between periodic images. Figure 2 summarizes the adsorption configurations considered in this work. Initial structures were generated by placing CH_3_OH, H_2_O, OH, and H species on Ti_5_c sites and bridging oxygen rows, followed by full structural relaxation. This approach enables systematic evaluation of the roles of co‐adsorbed water, hydroxyl groups, and hydrogen in stabilizing intermediates and modulating elementary steps relevant to methanol decomposition.

**FIGURE 2:**
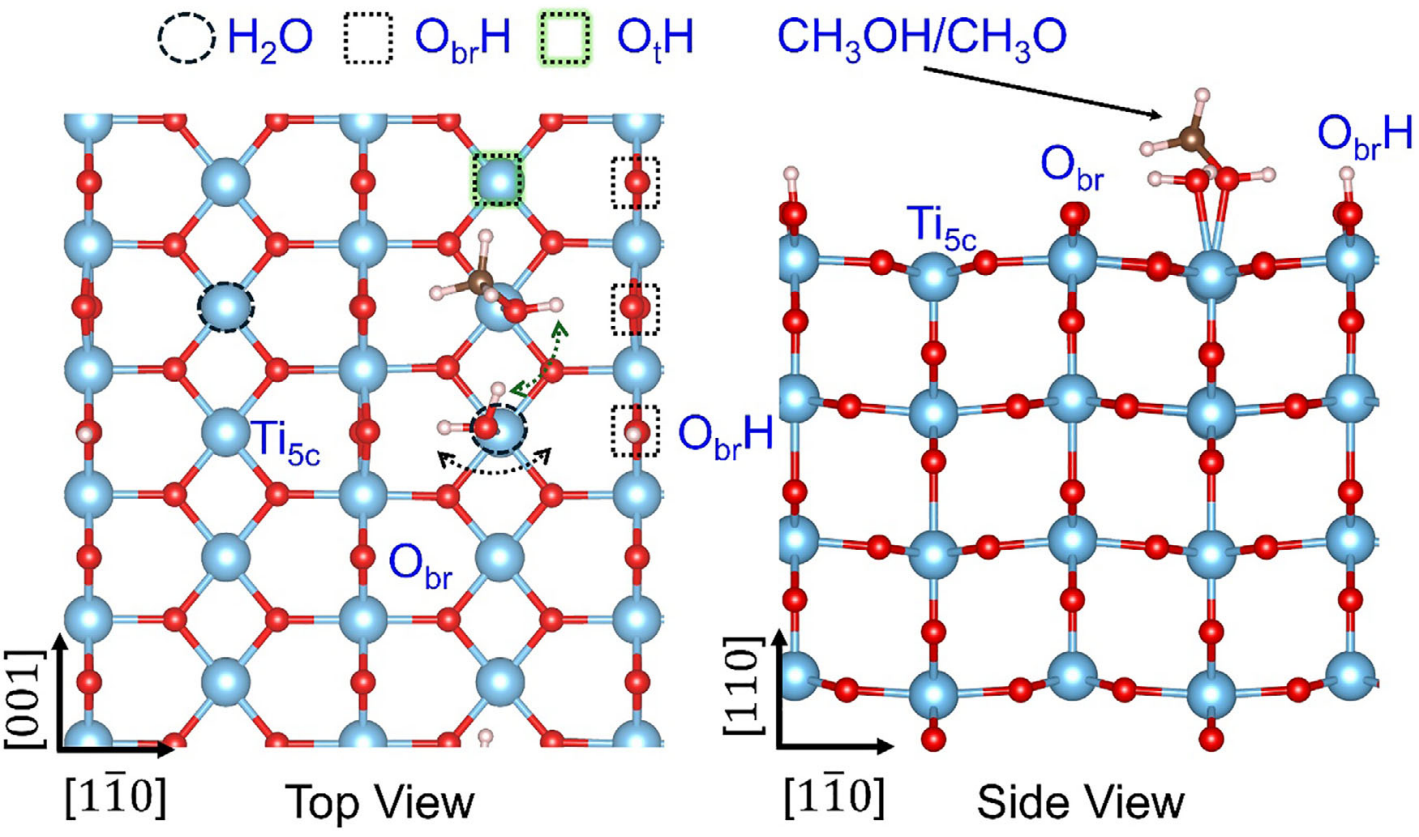
Supercell model of the R‐TiO_2_(110) surface. Top and side views along the [001] direction are shown in the left and right panels, respectively. For clarity, five‐coordinate titanium, bridging oxygen, terminal hydroxyl, and bridging hydroxyl species are denoted Ti_5_c, O_t_H, and O_br_H, respectively. The black dashed circles stand for the explored water adsorption sites (Figure S8 C1–C3). The black and green shadowed dashed squares stand for the explored O_br_H and O_t_H sites, respectively (Figure S11 C1–C3). While in preparation, the initial configurations of methanol and water could also be rotated along the green and black double‐arrowed direction, respectively, to change hydrogen‐bonding connection (such as Figure S8 C1–C2). On the site of methanol adsorption, a methoxy is placed to investigate the second deprotonation (Figure 6). Blue, red, white, and brown spheres represent Ti, O, H, and C atoms, respectively.

To support the assignment of the experimentally observed vibrational features, we first calculated the C─H stretching frequencies of adsorbed CH_3_OH and CH_3_O species using DFT. For methoxy, the calculated harmonic symmetric and asymmetric C─H stretching frequencies are 2995.1 and 3086.6 cm^−1^, respectively, whereas for methanol, the corresponding symmetric and asymmetric modes are located at approximately 3030.3 and 3143 cm^−1^, respectively. As expected within the harmonic approximation, these frequencies are systematically blueshifted relative to the corresponding experimental fundamental frequencies owing to the neglect of anharmonic effects. Nevertheless, the calculated frequencies reproduce the relative ordering of the symmetric and asymmetric C─H stretching modes and are consistent with the experimentally observed C─H stretching region. Further computational details and the complete vibrational‐frequency analysis are provided in (Figure S9 and Table S1).

To establish the reliability of our computational model for describing methanol adsorption and reaction energetics, we first examined methanol adsorption and the initial bond‐cleavage pathways on rutile TiO_2_(110). Methanol preferentially undergoes dissociative adsorption via O─H bond cleavage, yielding a surface methoxy species as the thermodynamically favored configuration. The calculated activation barrier for O─H dissociation is 0.32 eV, indicating a kinetically accessible process under ambient conditions. In contrast, C─H bond activation is significantly less favorable, with a barrier of 1.47 eV. These values are in good agreement with previous theoretical and experimental studies [21] supporting the robustness of the employed computational methodology. All subsequent analyses of environmental and interfacial effects were performed using the same computational parameters to ensure internal consistency across the reaction network.

To assess the role of co‐adsorbed water and hydrogen‐bonding in methanol decomposition, we first examined the influence of molecular water on the initial deprotonation step. In these calculations, a single H_2_O molecule was placed at a neighboring Ti_5_c site adjacent to adsorbed methanol. The calculated activation barrier for the first deprotonation (0.19–0.22 eV) is significantly lower than that on the pristine surface, indicating that even weakly interacting water molecules can efficiently promote proton transfer. The obtained barrier is in good agreement with recent reports on the water‐assisted activation of methanol on TiO_2_ [36]. Furthermore, when the hydrogen‐bond orientation of the co‐adsorbed water molecule is aligned on the same side as methanol, the barrier is reduced by an additional ∼0.03 eV, demonstrating that methanol deprotonation is highly sensitive to the local hydrogen‐bonding geometry.

The observed reduction in the activation barrier can be attributed primarily to indirect hydrogen‐bonding and electrostatic effects at the interface. Although the H_2_O molecule does not directly interact with the methanol O─H group, its hydrogen bond to a neighboring bridging oxygen atom stabilizes the proton‐accepting site and induces polarization of the local surface environment. This stabilization preferentially lowers the energy of the proton‐transfer transition state, thereby facilitating O─H bond cleavage and promoting dissociative adsorption.

We next introduced surface hydroxyl groups, which commonly form on TiO_2_ surfaces via water dissociation. In the presence of a bridging hydroxyl species, the methanol deprotonation barrier is further reduced relative to the pristine surface, reaching a minimum of ∼0.15 eV when the hydroxyl is located at the next‐nearest bridging oxygen site. Although the bridging hydroxyl does not directly participate in proton transfer, the excess electron associated with this species is localized in the vicinity of the bridging oxygen that accepts the proton from methanol. This proximity enhances stabilization of the transition state through local charge redistribution, suggesting a cooperative electronic effect that further lowers the reaction barrier.

The co‐adsorption of both water and hydroxyl species was subsequently examined to evaluate the combined influence of hydrogen‐bonding and excess charge. Under these conditions, the calculated barrier for the first methanol deprotonation decreases dramatically to ∼0.03 eV, indicating an essentially barrierless process (Figure 3). Moreover, when methanol is oriented toward an adjacent water molecule, an indirect dissociation pathway becomes accessible, in which proton transfer proceeds through the hydrogen‐bond network without the formation of a distinct high‐energy transition state. In all cases, the dissociated configuration is thermodynamically more stable than the intact molecular state.

**FIGURE 3:**
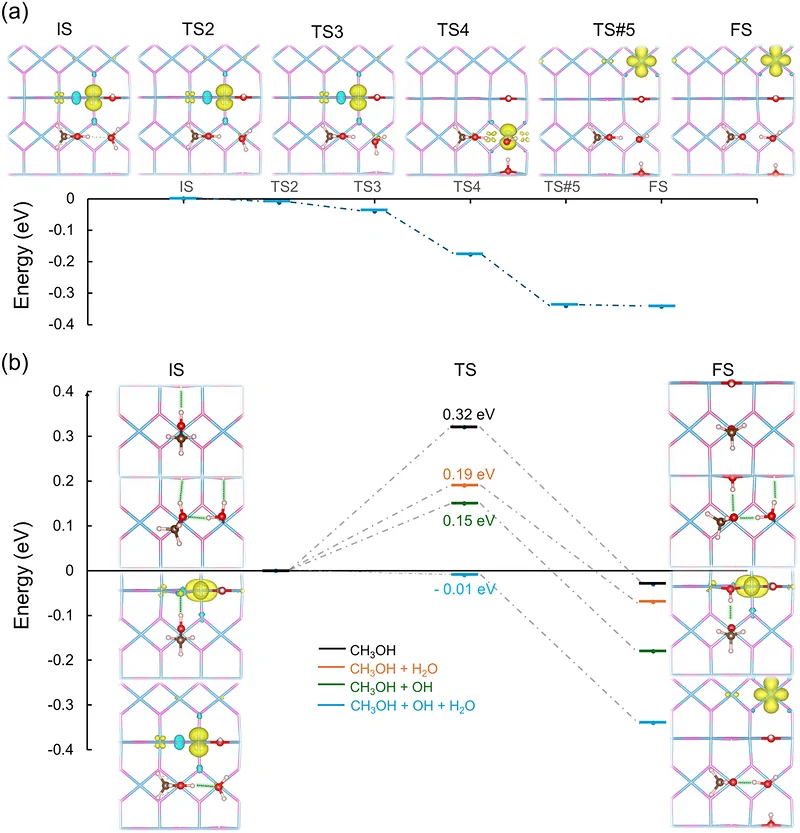
Minimum‐energy reaction pathways for the first deprotonation of methanol under different interfacial environments: (a) in the presence of co‐adsorbed H_2_O and bridging hydroxyl (O_br_H), and (b) representative additional adsorption configurations (only the lowest‐energy pathway for each case is shown). The activation barrier is substantially reduced in the presence of either H_2_O or OH and becomes effectively barrierless when both species are present at the surface. The blue and pink curves denote the substrate and adsorbates, respectively. All atoms directly involved in the deprotonation process are shown as spheres. Yellow and light‐blue isosurfaces represent spin density associated with the excess electron (unpaired localized electron) in the corresponding configurations, with an isovalue of 6 × 10^−^^3^. IS, TS, TS#, and FS denote the initial state, transition state, intermediate state, and final state along the reaction coordinate, respectively. TS denotes a transition state confirmed by the presence of one imaginary frequency, whereas TS# denotes an optimized local‐minimum intermediate with no imaginary frequencies.

The enhanced reactivity in this cooperative environment arises from many‐body hydrogen‐bonding interactions among multiple co‐adsorbed species. Within the short‐range network of water, hydroxyl, and methanol, collective hydrogen‐bonding stabilizes both the proton‐transfer transition state and the resulting dissociated configuration, leading to a systematic reduction in activation barriers. In addition, the presence of excess charge further facilitates the reaction dynamics. Along the computed pathway, the excess electron is initially localized near the bridging hydroxyl site, while a neighboring water molecule undergoes proton reorientation along the reaction coordinate. This structural reorganization preconditions the hydrogen‐bond network for charge redistribution and thereby promotes proton transfer.

Subsequently, the water molecule donates its proton to a nearby bridging oxygen, while the excess electron relocates to the underlying Ti_5_c site. The electron then migrates to another Ti_5_c site, further stabilizing the dissociated configuration and enabling the final methanol deprotonation step via an indirect mechanism. These results demonstrate that both the hydrogen‐bond network and localized excess charge act cooperatively to lower activation barriers and promote methanol decomposition under hydrated surface conditions.

Overall, the calculations reveal that methanol activation on hydrated oxide surfaces is governed not only by local bonding interactions but also by collective hydrogen‐bonding and charge‐stabilization effects. Such cooperative interactions provide a mechanistic basis for the enhanced reactivity observed experimentally under ambient and aqueous conditions, where water, hydroxyl species, and charge carriers coexist at the interface.

### 2.3 Time‐Resolved Evolution of Surface Methoxy Under Resonant Bandgap Photoexcitation

Figure 4a presents the temporal evolution of methoxy photooxidation following excitation at 400 nm with a 100 fs pulse duration. The SFG spectra reveal a well‐defined sequence of dynamical regimes governing the evolution of surface methoxy species. The spectral regions most strongly affected by photoexcitation are highlighted by dashed lines. An initial frequency shift, followed by pronounced intensity changes, indicates progressive methoxy depletion and the emergence of transient intermediates. Overall, the temporal response reflects distinct kinetic regimes associated with early‐stage structural reorganization and subsequent chemical transformation.

**FIGURE 4:**
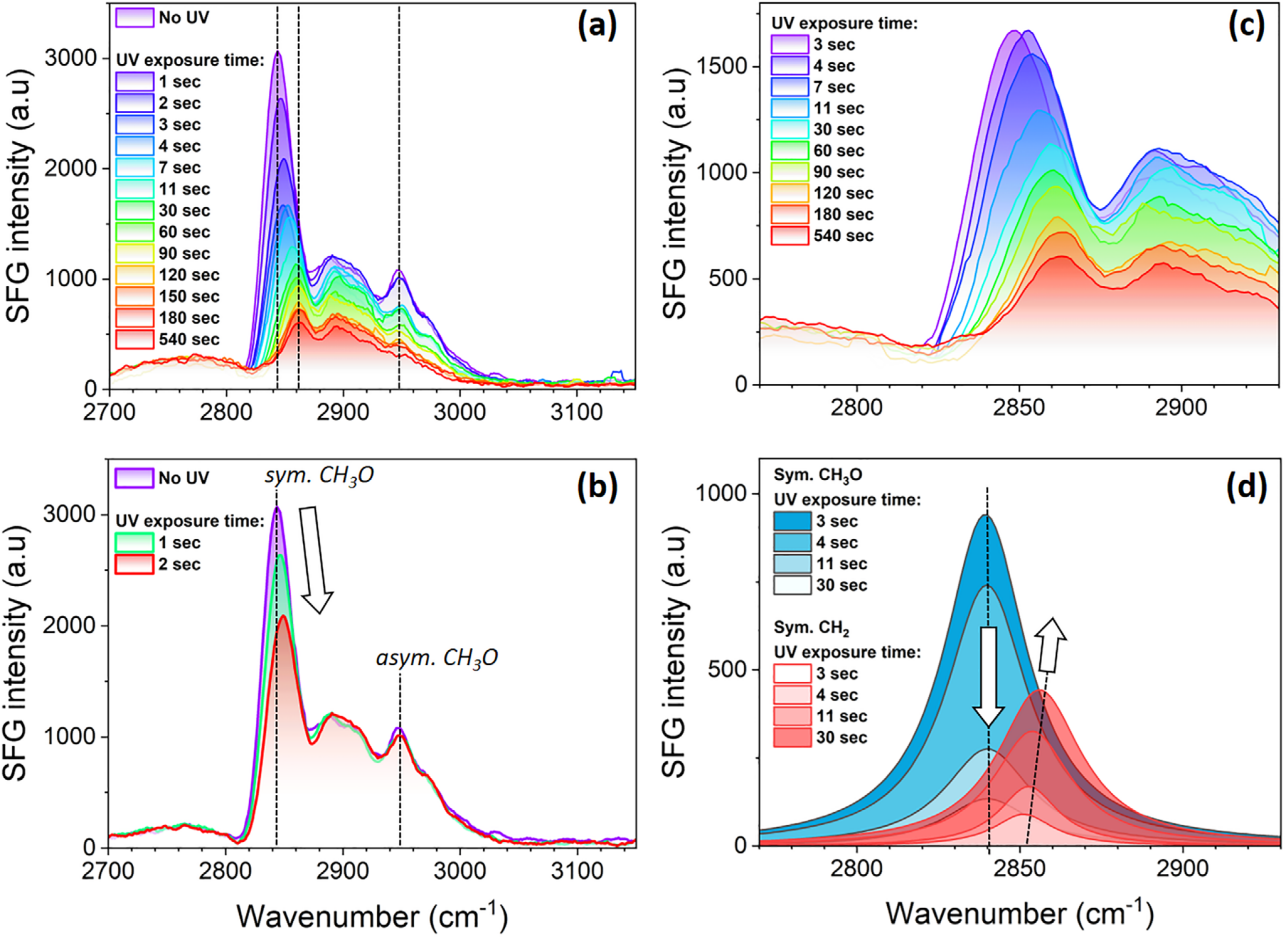
(a) Time‐resolved SFG spectra showing the evolution of methoxy photodecomposition under continuous excitation. (b) Early‐time spectra indicating orientational reorganization of methoxy prior to chemical transformation. (c) Methoxy photooxidation kinetics as a function of illumination time, leading to formation of a formaldehyde intermediate that subsequently converts to a stable surface hydroxymethyl species. (d) Spectral deconvolution demonstrating the conversion of methoxy (blue) to hydroxymethyl species (red), where the decrease in methoxy intensity coincides with the growth of the product signal. Fitted spectra at 4 and 30 sec are given in (Figure S7), where the necessity of two bands to accurately fit the spectra is discussed.

*Initial Regime: Orientational Reorganization (0.1–2 s)*: Within the first seconds of UV exposure, the SFG spectra show a gradual decrease in the intensity of the symmetric C─H stretching mode associated with CH_3_O species adsorbed at Ti_5_c, accompanied by a slight redshift in vibrational frequency (Figure 4b). In contrast, the antisymmetric stretching mode remains essentially unchanged in both intensity and spectral position during this interval. This selective spectral evolution indicates that the dominant process in the early stage is not chemical transformation but rather reorientation of methoxy species retained at Ti_5_c sites.

Because SFG intensity depends strongly on molecular orientation relative to the surface normal, a decrease in symmetric mode intensity without a corresponding loss of the antisymmetric signal is consistent with a redistribution of the methoxy tilt angle or azimuthal alignment. The concurrent frequency shift suggests a modification of the local chemical environment, likely arising from changes in intermolecular interactions, such as strengthened hydrogen‐bonding with neighboring surface hydroxyl groups or molecular water, rather than migration between adsorption sites. In this regime, the surface population of methoxy remains conserved at Ti_5_c sites, and the spectral evolution primarily reflects rapid orientational and structural relaxation following photoexcitation.

### 2.4 Intermediate State: Photooxidation and Formation of a New Surface Species

More pronounced spectral evolution becomes evident after approximately 3s of irradiation, as shown in Figure 4c. During this interval, the symmetric and antisymmetric stretching modes of CH_3_O─Ti_5_c decrease concurrently in intensity, accompanied by a distinct frequency shift between 3 and 4s. This shift signifies a change in the local coordination environment of surface methoxy species rather than simple population decay. At early irradiation times, methoxy is predominantly coordinated to stoichiometric Ti_5_c sites (Figure 1). Upon photoexcitation, reorientation of the adsorbate and restructuring of the interfacial hydrogen‐bonding network modify the local potential energy landscape, leading to a redistribution of stabilization from direct Ti─O coordination toward interactions with neighboring bridging oxygen rows. In this regime, methoxy remains formally bound to Ti_5_c sites but exhibits weakened Ti─O bonding and enhanced lateral stabilization through oxygen‐row interactions, consistent with a dynamically evolving adsorption geometry.

Concomitant with the decay of methoxy‐related bands, a new vibrational feature emerges at approximately 2850 cm^−1^ and increases progressively in intensity with irradiation time. As shown in Figure 4d, spectral deconvolution reveals a characteristic temporal buildup of this band, reaching a maximum at ∼30 s. This feature appears in the same spectral region as the symmetric CH3 stretching vibration of molecular methanol (∼2851 cm^−^^1^), as previously reported by SFG studies of methanol on TiO_2_(110), where the symmetric C─H stretching vibrations of molecular methanol and CH_2_‐containing species were shown to overlap, precluding an unambiguous assignment based on vibrational frequency alone [42]. Accordingly, although a contribution from molecular methanol cannot be excluded, its relatively weak SFG response and the spectral overlap prevent its reliable separation from the newly formed CH_2_‐containing species. Nevertheless, the real‐time SFG measurements clearly reveal the emergence and progressive growth of this new band, accompanied by the continuous decrease and progressive blueshift of the methoxy symmetric C─H stretching vibration under irradiation.

The correlated spectral evolution provides direct experimental evidence for the conversion of methoxy into new CH‐containing surface species during photooxidation. While the vibrational frequency alone does not uniquely identify the nature of the emerging intermediate, the observed temporal evolution is consistent with the formation of new surface intermediates along the photooxidation pathway.

### 2.5 Final State: Surface Stabilization and Further Oxidation

The newly formed surface species exhibits markedly slower photooxidation kinetics than methoxy under continuous illumination. The temporal evolution of its vibrational frequency and intensity, together with the corresponding evolution for methoxy, is summarized in Figure 5. In contrast to methoxy, which undergoes rapid initial reorientation followed by oxidative transformation, the intensity of the newly emerged feature, assigned to a CH_2_‐like species, remains nearly constant over the 30–100s time window under irradiation. This behavior indicates that the newly formed surface intermediate is more strongly bound to the surface and occupies a comparatively stable adsorption configuration.

**FIGURE 5:**
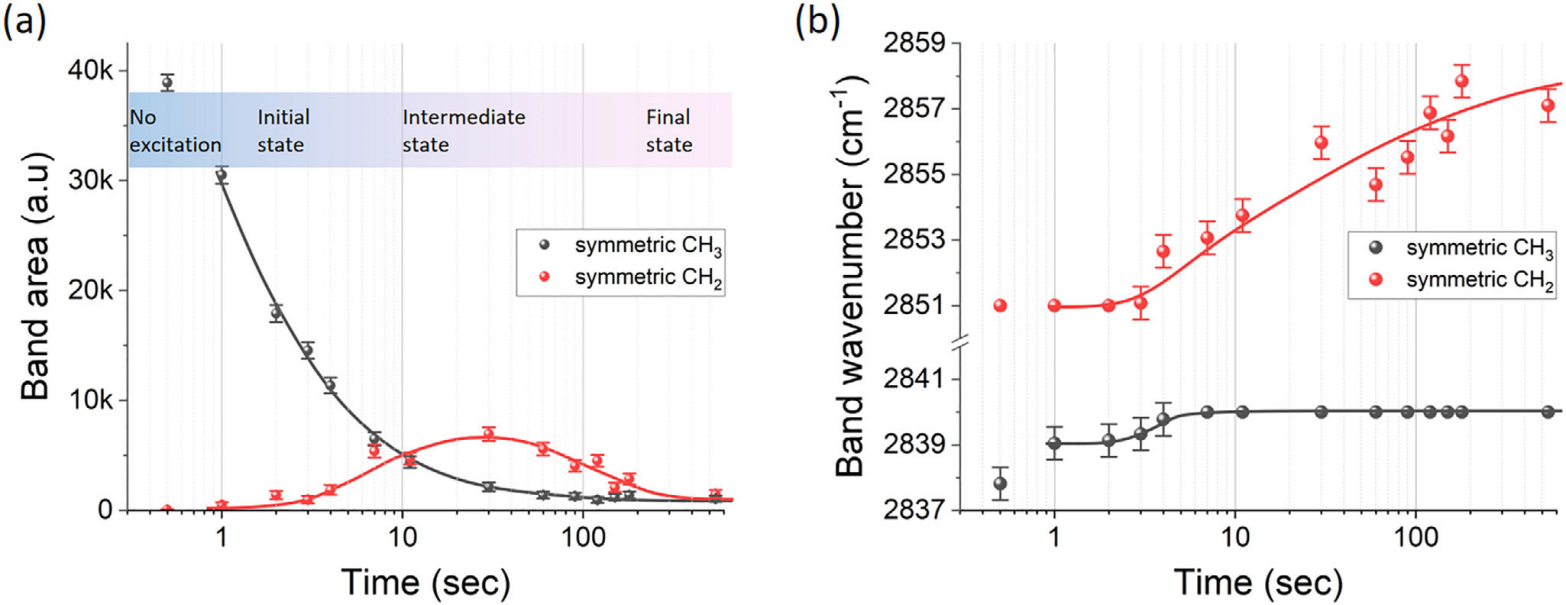
Time‐resolved kinetics of methoxy photooxidation under photoexcitation, showing the evolution of vibrational frequency and intensity as a function of irradiation time for both methoxy and the newly formed photooxidation product. The frequencies and area of both bands as well as their uncertainties (see SI.1.2 for details) were extracted from fits to the SFG spectra shown in Figure 4a. Continuous lines have been added for clarity. The frequency uncertainties are null for some spectra because the band positions were fixed to avoid inconsistent fitting.

A gradual decrease in intensity is observed only after ∼100 s, marking the onset of a slower oxidative conversion into more highly oxidized CH_x_ surface species. This delayed decay is consistent with the formation of a kinetically stabilized intermediate that persists on the surface prior to undergoing subsequent oxidation steps along the reaction pathway. Its specific chemical identity is discussed below in conjunction with the DFT reaction pathways and calculated vibrational frequencies.

### 2.6 DFT Calculations: Methoxy Oxidation Pathways and Intermediate Stabilization

To obtain molecular‐level insight into the reaction pathways inferred from time‐resolved spectroscopy, DFT calculations were performed to evaluate the energetics and structural evolution, and corresponding C─H stretching frequencies of methoxy species under photocatalytic conditions. The experimental results suggest that methoxy oxidation proceeds through sequential steps involving initial reorientation, site‐specific reactivity at Ti_5_c centers, and the formation of transient surface intermediates. In particular, the emergence of a new vibrational feature in the spectral region characteristic of the symmetric C─H stretching vibration of CH_2_‐containing intermediates is consistent with the formation of a transient CH_2_‐containing intermediate under photooxidative conditions [42].

Accordingly, the computational model considers a methoxy species adsorbed at a Ti_5_c site in the presence of neighboring H_2_O and OH species, representing a hydrated surface environment consistent with experimental conditions. The photoexcited state was approximated by introducing excess holes into the system. Test calculations indicated that the inclusion of excess electrons resulted in only minor changes to the calculated energetics of the elementary reaction pathway investigated here. Consequently, the excited‐state model focuses on the influence of photogenerated holes. This approximation is restricted to the static DFT description of the reaction energetics and does not imply that photogenerated electrons are unimportant in photocatalytic processes, where they play essential roles in charge recombination and reduction reactions. The resulting photooxidation pathway for methoxy in the presence of co‐adsorbed H_2_O/OH is shown in Figure 6a. In the early stage of the reaction pathway, the hole is initially localized at a bridging oxygen (O_br_) site. As the system evolves from the initial state toward the first transition state (TS5), hole migration away from the bridging oxygen is accompanied by reorientation of the methoxy species. This structural rearrangement is stabilized by hydrogen‐bonding interactions with nearby water molecules, consistent with the experimentally observed early‐time orientational dynamics under photoexcitation.

**FIGURE 6:**
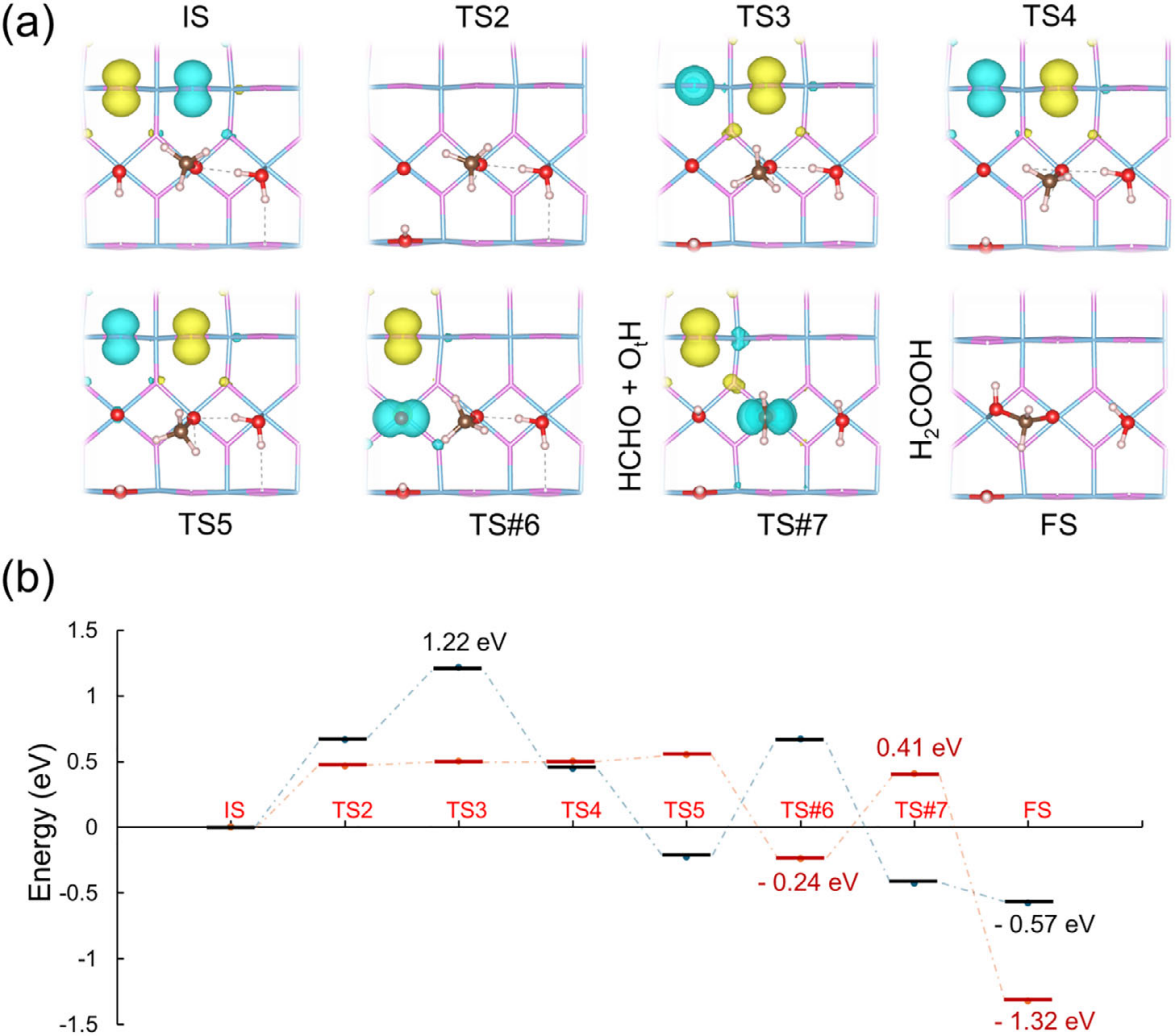
(a) Calculated methanol decomposition on hydrated TiO_2_(110) in the presence of a mixture of H_2_O and OH species. The yellow and blue isosurfaces represent the spin density of unpaired localized holes (two holes) in the corresponding configurations. In some cases, the unpaired holes are absent, indicating that all excess holes are either paired or delocalized. The isovalue is set to 6 × 10^−^^3^. (b) Minimum energy path profiles for the second deprotonation of methoxy: (Red) in the presence of H_2_O/O_t_H and (black) with only O_t_H. IS, TS, TS#, and FS denote the initial state, transition state, intermediate state, and final state along the reaction coordinate, respectively. For the system without water (black curve), TS5–TS7 are confirmed transition states.

Further progression along the reaction coordinate leads to localization of the hole at a terminal oxygen site (TS6), which facilitates a second deprotonation step of the methoxy species. This process yields an intermediate state comprising a formaldehyde‐like (HCHO) fragment and a surface hydroxyl (O_t_H) group. The calculated activation barrier for this photooxidation step is ∼0.64 eV (Figure 6b, red curve), significantly lower than the barrier for C─H bond cleavage of methoxy on a clean surface under steady‐state conditions (∼1.47 eV). This substantial reduction in activation energy underscores the cooperative role of the hydrated environment and localized holes in promoting methoxy activation and subsequent oxidation.

Although the HCHO and O_t_H intermediate configuration is thermodynamically less stable than the initial methoxy state, the system subsequently undergoes recombination to form a hydroperoxymethyl (H_2_COOH) surface species. This final state (FS) is substantially more stable than the initial configuration, with an energy stabilization of approximately 1.32 eV relative to the methoxy state. The formation of hydroperoxymethyl therefore represents a thermodynamically favorable endpoint in the early stages of methoxy photooxidation on the hydrated surface.

To further assess the role of water in the reaction mechanism, an alternative reaction pathway was examined in which the same initial methoxy configuration was considered in the absence of neighboring water molecules. Under these conditions, the reaction pathway changes markedly (Figure 6b, black curve). Most notably, the calculated activation barrier for methoxy deprotonation increases to approximately 1.22 eV, nearly doubling relative to that in the hydrated system. In addition, localized hole states disappear once the O_t_H species loses its proton, indicating that the absence of water destabilizes surface hole localization.

To evaluate whether the proposed reaction pathway is consistent with the experimental SFG observations, harmonic C─H stretching frequencies were calculated for the key reaction intermediates (see Figure S9 and Table S1). The calculated frequencies for H_2_COOH fall within the spectral region previously assigned to the symmetric C─H stretching vibration of CH_2_‐containing species and are consistent with the experimentally observed band near 2850 cm^−1^. Together with the favorable reaction energetics, these calculations provide additional support for assigning the emerging vibrational feature to H_2_COOH formed during methoxy photooxidation.

These results demonstrate that interfacial water plays a critical role in stabilizing excess holes through hydrogen‐bond‐mediated charge localization, thereby lowering the activation barrier for methoxy oxidation. The computed methoxy reorientation in the hydrated, hole‐rich environment is consistent with the experimentally observed early‐time decrease in SFG intensity prior to significant methoxy depletion. Moreover, the predicted formation of the hydroperoxymethyl (H_2_COOH) species provides a plausible structural origin for the transient CH_2_‐related vibrational feature observed during the intermediate time regime.

These findings contrast with recent near‐ambient‐pressure studies [43] of methanol on TiO_2_(110), where the absence of detectable methoxy oxidation under UV illumination was attributed to recombinative desorption of bridging hydroxyl O_br_H species as water, thereby regenerating bridging oxygen vacancy (Ov) sites that sustain continuous dissociative adsorption of methanol and persistent methoxy formation. In contrast, under ambient conditions explored here, interfacial water and hydroxyl networks actively participate in hydrogen‐bonding and charge‐localization processes that promote, rather than suppress, methoxy transformation.

Collectively, the experimental and computational results support a mechanism in which methoxy photooxidation proceeds through hole‐assisted activation involving surface oxygen atoms and subsequent intermediate formation pathways that are strongly modulated by interfacial water. The calculations show that photogenerated holes initially localize on O_br_ sites before charge redistribution toward the adsorbed methoxy species, suggesting that transient oxygen‐centered reactive species may contribute to the oxidation process [14, 32]. Hydrogen‐bonded water molecules stabilize localized holes, reduce reaction barriers, and thereby enable efficient photocatalytic oxidation at Ti_5_c surface sites. Although the present results do not allow us to distinguish unambiguously between direct hole transfer and indirect oxidation mediated by transient oxygen‐centered species (e.g., O^−^ or •OH), both pathways are consistent with the calculated charge localization and the observed photocatalytic behavior under hydrated conditions.

## 3 Conclusion

In this work, we present a molecular‐level investigation of methanol adsorption, activation, and photooxidation on hydrated rutile TiO_2_(110) under ambient conditions. Surface‐specific SFG spectroscopy shows that methanol deposited from liquid solution predominantly forms methoxy species bound to Ti_5_c sites, confirming that dissociative adsorption is the dominant pathway at the hydrated oxide–liquid interface. Complementary DFT calculations reveal that co‐adsorbed water and surface hydroxyl groups play a decisive role in facilitating methanol activation by stabilizing the proton‐transfer transition state and reducing the barrier for O─H bond cleavage to near‐barrierless values. Under photoexcitation, time‐resolved SFG measurements resolve sequential reaction regimes involving methoxy reorientation, chemical transformation, and intermediate formation. DFT calculations identify hole‐assisted oxidation pathways that yield a hydroperoxymethyl final stable surface species, consistent with the observed transient CH_2_ vibrational signatures. The calculations further show that hydrogen‐bonded interfacial water stabilizes localized hole states and lowers the activation barrier for methoxy oxidation, underscoring the central role of the hydrated interface in governing photocatalytic reactivity.

These results demonstrate that methanol activation and oxidation on TiO_2_ under realistic environmental conditions are governed by cooperative interactions among interfacial water, surface hydroxyl groups, and photogenerated charge carriers. By revealing how hydration enables near‐barrierless proton transfer and stabilizes hole‐driven oxidation pathways, this work provides a mechanistic framework for alcohol oxidation at oxide–liquid interfaces and highlights interfacial hydrogen‐bond networks as a key design parameter for photocatalytic systems operating under ambient conditions.

## Author Contributions

**Milad Salarinasab**: investigation and formal analysis. **Julian Andreas Hochhaus**: investigation and formal analysis. **Jing Wang**: software and formal analysis. **Aimeric Ouvrard**: formal analysis and investigation. **Anas Akhtar**: investigation. **Michele Capra**: investigation. **Kristina Tschulik**: formal analysis and investigation. **Carsten Westphal**: investigation and formal analysis. **Mirko Cinchetti**: investigation and formal analysis. **Bo Wen**: formal analysis and writing – original draft. **Li‐Min Liu**: formal analysis. **Zhe Wang**: formal analysis. **Ahmed Ghalgaoui**: conceptualization, supervision, funding acquisition, writing ‐ original draft, and validation.

## Conflicts of Interest

The authors declare no conflicts of interest.

## Supporting information



The Supporting Information contains additional experimental and theoretical details, 14 figures, and 17 supplementary references.**Supporting File**: anie74576‐sup‐0001‐SuppMat.docx.

## Data Availability

The data that support the findings of this study are available from the corresponding author upon reasonable request.
