# Supplementary material for: Methanol Decomposition on TiO2 Under Ambient Conditions: Cooperative Proton Transfer at Hydrated Interfaces
Source: Angew Chem Int Ed Engl. 2026 Sep 7;65(46):e3448039. doi: 10.1002/anie.3448039 (PMC13749320; doi:10.1002/anie.3448039)
Supplement: Supplementary file 1 [file ANIE-65-e3448039-s001.docx]

**Supporting information**

**Methanol Decomposition on TiO₂ under Ambient Conditions: Cooperative Proton Transfer at Hydrated Interfaces**

Milad Salarinasab^[a]^, Julian Andreas Hochhaus^[a]^, Jing Wang^[b]^, Aimeric Ouvrard^[c]^ , Anas Akhtar^[d]^, Michele Capra^[a]^, Kristina Tschulik^[d]^, Carsten Westphal^[a]^, Mirko Cinchetti^[a]^, Bo Wen* ^[b]^, Li-Min Liu^[e]^, Zhe Wang^[a]^, and Ahmed Ghalgaoui *^[a]^

[a]	Milad Salarinasab, Dr. Julian Andreas Hochhaus, Dr. Michele Capra, Prof. Carsten Westphal, Prof. Mirko Cinchetti, Prof. Zhe Wang, Priv.-Doz. Dr. Ahmed Ghalgaoui
Department of Physics
TU Dortmund University
Otto-Hahn-Straße 4, 44227 Dortmund, Germany
E-mail: ahmed.ghalgaoui@tu-dortmund.de

[b]	Dr. Jing Wang, Prof. Bo Wen
School of Physics and Electronics, Henan University, Kaifeng, China
Email: bo.wen@henu.edu.cn

[c]	Dr. HDR. Aimeric Ouvrard
Institute des Sciences Moléculaires d’Orsay (ISMO), Paris Saclay University, 598 Rue André Rivière, 91400 Orsay, France

[d]	Anas Akhtar, Prof. Kristina Tschulik
Chair of Analytical Chemistry- Electrochemistry and Nanoscale Materials, Ruhr-Universität Bochum, Universitätsstrasse 150, 44801 Bochum, Germany

[e]	Prof. Li-Min Liu
School of Physics, Beihang University, Beijing, China

# S1. Sum-Frequency Generation Spectroscopy

## S1.1 Experimental Setup

SFG measurements were performed using a regeneratively amplified femtosecond Ti:sapphire laser system (Coherent), delivering 800 nm pulses (~100 fs, 1 kHz). A portion of the fundamental output was directed into an optical parametric amplifier followed by a difference-frequency generation stage (AgGaS2 crystal) to produce tunable mid-infrared radiation spanning 2700–3100 cm^−1^, covering the C–H stretching region of methanol and its photodecomposition products1. The remaining 800 nm output served as the visible up-conversion beam. The visible and mid-infrared beams were directed onto the sample surface at incidence angles of 53° and 48° from the surface normal, respectively, and spatially overlapped in a co-propagating reflection geometry. At the sample position, the visible and mid-infrared beams delivered approximately 26 mW and 24 mW average power, respectively, with spot diameters of ~2 mm. All spectra were acquired in the PPP polarization configuration. Photodecomposition was induced by 400 nm UV radiation (~25 mW, ~3 mm spot diameter), generated by frequency-doubling the 800 nm beam in a β-barium borate crystal and directed onto the sample at near-normal incidence for controlled durations of 0–540 s. A schematic of the optical layout is shown in Figure S1.

The SFG signal was collected in the specular reflection direction, spatially filtered, and passed through a short-pass filter to reject residual 800 nm light. The filtered signal was dispersed by spectrograph (Andor Shamrock) onto a thermoelectrically cooled CCD detector (Andor iDus), providing a spectral resolution of approximately 4 cm^−1^. For each condition, a signal spectrum was first acquired from the methanol-treated TiO_2_(110) surface, followed by a background measurement obtained by blocking the visible beam under otherwise identical optical conditions.

Wavenumber (cm-1)

**Figure S1.** Schematic of the vibrational SFG spectrometer. The 800 nm fundamental beam is split at the beam splitter (BS) into a visible arm for SFG up-conversion and a pump arm driving the optical parametric amplifier (OPA) and non-collinear difference frequency generation (NDFG) module for tunable mid-infrared generation. The two beams propagate through independent optical paths — directed and steered by gold mirrors (GM1–GM9) and protected silver mirrors (M0–M11) — and overlap directly on the sample surface in a co-propagating reflection geometry. Beam alignment and spatial filtering are assisted by an iris (I), lens (L), and diffraction grating (G) which used for pulse shaper in visible path. The generated SFG signal is collected and analyzed using a spectrograph equipped with a CCD detector (Detector), with unwanted beam contributions rejected by beam blockers (Blocker). green lines indicate the 800 nm pump beam path; red lines indicate the mid-infrared beam path.

## S1.2 Data Analysis and Spectral Fitting

Raw SFG spectra were processed using custom Labview scripts. The background spectrum was subtracted from each signal spectrum to isolate the sample contribution. Adjacent averaging algorithm (5 points large window) was used to slightly smooth the data. Detected SFG wavelengths (λ_SFG_) were converted to infrared wavenumbers via the relation ν̃_IR_ = 10^7^/λ_SFG_ − 10^7^/λ_VIS_ (λ_VIS_=795 nm). Cosmic ray artifacts were removed using a Savitzky-Golay filter.

Spectral fitting was performed using Eq. 1, where the SFG intensity is proportional to the squared magnitude of the effective second-order nonlinear susceptibilities of the bare surface (non-resonant contribution) and the adsorbed molecules, where, A_q_, ω_q_ , Γ_q_ and φ are the amplitude, center frequency, linewidth and the relative phase between the resonant and non-resonant contributions. The susceptibility of a constant was modeled as the sum of a non-resonant background and Lorentzian resonant contributions, respectively. The parameters were determined using a nonlinear least-squares minimization unweighted Levenberg-Marquardt algorithm.

*I_SFG_* ∝ |*χ*^(2)^*_eff_*|^2^ = |*A_NR_ e^iφ^* + Σ*_q_* *A_q_* / (*ω_IR_* − *ω_q_ + iΓ_q_*)|^2^	(1)

The spectral fits were rigorously performed to ensure the robustness of our interpretations. We proceeded in successive stages, supported by performance and consistency tests across the entire dataset, to enable the most precise and reliable determination of the physical parameters and their evolution during photo-induced changes. At the first step, only the most obvious bands are fitted as well as IR shape, non-resonant intensity and the phase difference. For each series, we start from the first spectrum, then we use the resulting fitting parameters as initial parameters for the next spectrum of the time-dependent UV irradiation series. It ensures a fast fit convergence, smooth transitions and avoids unphysical changes from spectrum to spectrum. Fitting of the whole series is then restarted after fixing parameters that remained almost constant (IR bandwidth, IR position, the bandwidth of weak vibration) over the series or additional band are included when a single band can obviously not fit a part of the spectrum. An example is given in SI.6 where the fit quality is compared when using one or two bands at the transition from the methoxy band to CH_2_ band under UV illumination between 4 and 30 sec. The phase difference between NR and bands must be constrained at 0.2 rad to avoid additional band frequency shift.In Eq. 1, since χ_NR_ is a constant, the non-resonant contribution in the SFG spectrum is the product of (χ_NR_)²*IR(σ). Consequently, the NR contribution reflects the IR spectral shape. The latter can easily be fitted by a 160 cm^-1^ full-width at half-maximum Gaussian shape centered at 2870 cm^-1^ on the last spectra of the series, where vibrational contributions are weaker. The IR shape was also accurately fitted for all UV illumination times by fitting accurately the wings of the NR contribution far from vibrational bands in the 2650-2800 cm^-1^ range. Fitting freely the IR position (2870 cm^-1^) and bandwidth (160 cm^-1^) did not show significant drift of those parameters as expected for normal laser operation. Therefore, IR position and bandwidth were kept constant for the whole series, as their relative small changes is granted by the high laser spectral stability. The IR shape was also confirmed of a reference sample of GaAs(111), that shows a similar IR position, but slightly broaden on the high energy side because of a volume contribution of GaAs that has a non-centrosymmetric crystalline structure.

Along fitting cycles, it was observed that fitting freely the phase for all spectra led to a moderate drift from 0.2 to 0.6 rad inducing a shift of -2 cm^-1^ in all band positions. In absence of physical justification for the phase shift and considering the impact on band position, even small, the phase was constrained at 0.2 rad. We observed a constant decrease of the χ_NR_ intensity under illumination. Indeed, the NR is known to be sensitive to molecular coverage and composition as the molecular hybridization to surface density of states directly affects the non-linear susceptibility of the surface.

# The precision on the band position extracted from the fit and presented in Figure 5, is limited by the confidence interval (around 0.3 cm^-1^), itself limited by the signal to noise ratio that is quite low.

# S2. Sample Preparation

Rutile TiO_2_(110) single crystals (MaTeck GmbH) were used, oriented to within 0.1° of the (110) crystallographic plane. The samples were prepared in an ultrahigh vacuum (UHV) chamber by repeated cycles of Ar^+^ ion sputtering followed by thermal annealing. Heating was performed via thermal radiation from a tungsten (W) filament positioned behind the sample. The system was temperature-calibrated using a thermocouple directly attached to the sample holder at the backside of the single crystal.

As established in the literature^[1–3]^, TiO2(110) exhibits two distinct surface reconstructions depending on the annealing temperature. Annealing up to 900 K results in a (1×1) unreconstructed surface, as confirmed by low-energy electron diffraction (LEED). At higher temperatures (~980 K), the LEED pattern exhibits characteristic streaks along the $\left[ 1\overline{1}0 \right]$ direction. When the annealing temperature exceeds ~1120 K, these features evolve into a distinct (1×2) diffraction pattern. For all samples investigated in this study, an annealing temperature of 900 K was selected to consistently produce the unreconstructed (1×1) surface.

The preparation procedure consisted of the following sequential steps. The sputtering step was performed for 15 minutes under an argon atmosphere of 4×10−6 mbar, rastering an area of 6×6 mm^2^ with an ion current density of 1 μA/cm^2^ at the sample surface. Following sputtering, the annealing procedure comprised three stages: a ramp-up phase lasting 80 minutes during which the temperature was increased following a parabolic function under the same argon atmosphere, a steady heating phase at the target temperature for 30 minutes, and a ramp-down phase of 80 minutes during which the temperature was decreased parabolically. The parabolic ramping profile was chosen so that close to the desired annealing temperature, both during ramp-up and ramp-down, the temperature step becomes very small (~2 °C/min), thereby reducing thermal stress and preventing cracking of the crystal.

Following surface preparation in UHV, each TiO_2_(110) crystal was removed from the vacuum chamber and immediately immersed in anhydrous methanol (Sigma-Aldrich, 99.8%, stored over molecular sieves) for 12 hours at room temperature. This extended exposure ensured saturation coverage of methanol on the surface. After immersion, the samples were removed from the methanol bath, briefly dried under a stream of dry nitrogen, and promptly transferred to the SFG measurement setup.

# S3. LEED Characterization

The surface structural quality and reconstruction of the TiO_2_(110) crystals were verified by low-energy electron diffraction (LEED) prior to methanol adsorption. LEED patterns were recorded at multiple electron kinetic energies to confirm the surface periodicity and assess long-range order.

## S3.1 Temperature-Dependent Surface Reconstructions

Figure S2 illustrates the structural evolution of the TiO2(110) surface as a function of annealing temperature, as observed by LEED. At annealing temperatures up to 900 K, the surface exhibits a well-defined (1×1) diffraction pattern characteristic of the unreconstructed, near-stoichiometric surface. As the annealing temperature is increased to approximately 980 K, the LEED pattern develops characteristic streaks along the $\left[ 1\overline{1}0 \right]$ azimuth, indicative of incipient surface restructuring. At temperatures exceeding ~1120 K, these streaky features sharpen into discrete fractional-order spots consistent with a (1×2) reconstruction, which is attributed to the formation of Ti2O3 added rows or extensive ordering of reduced surface structures^[1]^.

**Figure S2.** Structural evolution of the rutile TiO_2_(110) surface as a function of annealing temperature observed by LEED. The (1×1) unreconstructed surface is obtained at annealing temperatures up to 900 K. Streaky features appear at approximately 980 K, evolving into a well-defined (1×2) reconstruction above ~1120 K.

## S3.2 Comparison of (1×1) and (1×2) Surface Reconstructions

Figure S3 presents a direct comparison between the (1×1) and (1×2) surface reconstructions observed via LEED, shown alongside kinematic simulations of the diffraction patterns calculated using LEEDPat software^[4]^. Both experimental reconstructions yield sharp diffraction patterns, demonstrating high long-range and local order. The experimental patterns show excellent agreement with the expected (1×1) and (1×2) surface periodicities, confirming the reliability of the preparation protocol. The (1×1) pattern displays only integer-order diffraction spots, whereas the (1×2) pattern exhibits additional half-order spots along the [001] direction, reflecting the doubled real-space periodicity perpendicular to the bridging oxygen rows.

**Figure S3.** Structural analysis of rutile TiO2(110) surface reconstructions. Experimental LEED patterns for the (1×1) and (1×2) reconstructions are shown alongside their corresponding kinematic simulations calculated using LEEDPat^[4]^. The agreement between experiment and simulation confirms the expected surface periodicities.

# S4. Atomic Force Microscopy Characterization

The surface topography of the prepared ritile TiO_2_(110) crystal exhibiting the (1×1) reconstruction was characterized by atomic force microscopy (AFM) to assess the surface morphology, terrace structure, and roughness at the nanometer scale.

## S4.1 Instrument and Measurement Parameters

AFM imaging was performed using a Bruker BioScope Resolve^TM^ system operated in PeakForce Quantitative Nanomechanical Mapping (PF-QNM) mode under ambient conditions (air) at room temperature. A ScanAsyst-Air probe from Bruker was employed, featuring a spring constant of 0.4 N/m, a nominal tip radius of 2 nm, a resonance frequency of 70 kHz, and a triangular cantilever geometry. The measurements were performed with a 2 kHz Peak Force resonant frequency, a scan rate of 1 Hz, and a resolution of 256 × 256 pixels. The peak force setpoint was set to 1.4 nN with a peak force amplitude of 120 nm. Image analysis and processing were carried out using NanoScope Analysis 3.0 software.

## S4.2 Surface Topography

Figure S4a presents an AFM height image of the rutile TiO_2_(110) (1×1) surface acquired over a 1 × 1 μm^2^ scan area. The image reveals a well-defined terrace-and-step morphology characteristic of a properly prepared single-crystal oxide surface. The terraces appear as relatively flat, extended regions separated by step edges running approximately parallel to the [001] crystallographic direction. The total height variation across the imaged area is approximately 2.5 nm, indicating that the surface consists of several atomically flat terraces separated by monoatomic or diatomic steps. The terrace widths range from approximately 50 to 200 nm, consistent with the nominal miscut angle of the crystal.

Figure S4b shows a higher-magnification AFM height image acquired over a 500 × 500 nm^2^ scan area on the same sample. At this magnification, the individual terraces and step edges are more clearly resolved. The terraces appear smooth and uniform, with large-scale roughening. The step edges are generally straight with occasional kink sites, which is consistent with thermodynamic equilibration achieved during the annealing process. The observed surface morphology confirms that the preparation procedure produces a clean, well-ordered TiO_2_(110) surface suitable for subsequent methanol adsorption and spectroscopic investigation.

**Figure S4.** AFM height images of the rutile TiO2(110) (1×1) surface acquired in PeakForce QNM mode under ambient conditions. (a) Large-area scan (1 × 1 μm^2^) showing the terrace-and-step morphology with a total height range of 2.5 nm. (b) Higher-magnification scan (500 × 500 nm^2^) of the same surface, revealing smooth, flat terraces separated by well-defined step edges. The scale bars correspond to 200 nm and 100 nm, respectively.

# S5. XPS measurements of TiO_2_(110) following exposure to air

Figure S5. XPS spectra of the O1s band of R-TiO_2_(110) under UHV (a), after air exposure (b) and after methanol exposure (c).

The hydration of R-TiO_2_(110) upon air exposure was investigated using XPS. Figure S5 presents the O1s XPS spectra of R-TiO₂(110) measured under UHV conditions and after air exposure. Under UHV, only a single peak centered at 530.5 eV is observed, corresponding to lattice oxygen in TiO₂. The slightly asymmetry of the O1s peak is related to the presence of surface defects induced by the cleaning process in UHV, namely cycles of sputtering and annealing^[5]^. After exposure to air and subsequent reintroduction into UHV, the O1s spectrum exhibits an additional shoulder centered at 532.2 eV alongside the main peak, compatible with the formation of surface hydroxyl species. This observation is consistent with previous reports on hydroxylation of TiO₂(110) surfaces upon exposure to ambient conditions.

After exposure to methanol (Fig. S5c), two additional contributions are observed in the O1s region. We first note that the main oxygen peak associated with the TiO_2_(110) crystal shifts by approximately 0.5 eV toward lower binding energy after methanol exposure and is now located at 530.0 eV. This shift may be related to changes in the surface electronic structure induced by methanol adsorption and/or to the oxidation state of the TiO_2_ surface following its prior exposure to air. It is well known that UHV preparation of TiO_2_(110) may lead to a reduced surface, whereas subsequent air exposure can compensate for this reduction.

The two additional peaks, located at 530.6 eV and 531.3 eV, are consistent with the formation of hydrated hydroxyl species (OH–H_2_O)^[6]^ and C=O-containing species^[7]^, respectively. It is worth noting that the deconvolution of the O 1s region in the presence of hydroxyl- and carbonyl-containing species is challenging, as the binding energies of these species may overlap.

# S6. Additional SFG measurement and fitted spectra

To distinguish the spectral contributions of adsorbed methanol from adventitious surface contamination, SFG spectra of the rutile TiO₂(110) surface were recorded with and without methanol treatment under identical optical conditions. Figure S6 compares the bare surface spectrum (red) with the methanol-treated surface (black) in the C–H stretching region. The air-exposed sample prepared under UHV conditions exhibits weak vibrational features associated with adventitious hydrocarbons and trace acidic contaminants commonly present after ambient exposure. In contrast, methanol deposition produces a substantially stronger absolute vibrational response, particularly near 2835 cm^-1^, consistent with the formation of methanol-derived surface species. The pronounced signal enhancement confirms that the observed spectral features are dominated by adsorbed methanol rather than residual hydrocarbon contamination.

**Figure S6.** SFG spectra in the C–H stretching region of the TiO₂(110)-(1×1) surface recorded before methanol exposure and after methanol adsorption. The initial surface (pre-exposure) is shown in red, and the spectrum after methanol desorption is shown in black.

To convince the reader of the appearance of a new band at 2851 cm^-1^ as shown in Figure 5, we plot in Figure S7, the fitted spectra at 4 et 30 sec using one or two bands. Residues are compared to highlight the better quality of the fit using two bands.

# Figure S7. Deconvoluted SFG spectra at 4 an 30 sec of UV irradiation time where one or two bands were used to fit the data.

# S7. DFT Calculations

To explore the influence of ambient environment, theoretical calculations are performed using density functional theory (DFT) as implemented in the CP2K software package^[8]^, which applied a plane-wave and Gaussian hybrid basis scheme^[9]^. Molecular optimized double zeta polarized m-DZVP basis were used for the Gaussian part, and a cutoff of 320 Ry was adopted for the plane waves. The interaction between core and valence electrons was treated by Coedecker-Teter-Hutter (GTH) norm-conserved pseudopotentials^[10]^. In addition, Perdew-Burke-Ernzerhof (PBE) functional^[11,12]^was employed to account for the exchange and correlation interaction between valence electrons. Additional electrons (or holes) are introduced by adsorbing H (OtH, CH3O) radicals, which play as electron donor (acceptor) defects. To better describe the electronic band gap, the onsite Coulomb interaction correction was added using a Hubbard model, where U = 3.5 (8.8) eV for Ti-3d (O-2p)^[13]^. To localize the excess charges, both Ti-3d and O-2p orbitals are applied on-site U correction. The Grimme DFT-D3 method^[14]^ was adopted to include the long-range van der Waals interaction. Relaxation was realized while the residual forces on all atoms are less than 0.05 eV/Å and single Γ point was sampled for the reciprocal space. To evaluate the reactions’ barrier of methanol decomposition, the climbed-image nudged-elastic-band (CI-NEB)^[15]^ calculations were also carried out to determine the transition states and reaction path energy profiles. The substrate was modeled as an unreconstructed rutile TiO₂(110) 4 × 2 surface slab consisting of four trilayers, separated by a ~15 Å vacuum layer to eliminate periodic interactions.

Figure S8 gives the various structures of isolated methanol and isolated water molecule mix adsorption. Here, the possible configurations are considered to check their stability. For instance, water molecules could be placed near the methanol on the Ti_5c_ site (C1-C3 and C6) or via H-bonding (C4 and C5). While on Ti_5c_ site, the water could also H-bond with bridging O through different orientations (C1-C3) or even direct to the methanol (C6). However, the adsorption of H-bonding approach is less favorable (~ 0.25 eV larger in total energy), they were not considered further for deprotonation investigations. Moreover, the second layer water adsorption may induce an indirect methanol dissociation as reported in^[16]^ which is beyond the scope of this work, where mainly the methanol dissociation at low coverage water adsorption is compared. Therefore, the effect of multiple layer water adsorption will be discussed in an expanded work separately.

**Figure S8**: Considered methanol and water mix adsorption configurations and their relative energy. Configurations marked with a blue ‘X’ were excluded from further calculations.

DFT calculations were performed to determine the C–H stretching frequencies of the relevant adsorbed species, namely methanol (CH₃OH), methoxy (CH₃O), and the proposed partially dehydrogenated intermediate CH₂OOH. The calculated vibrational frequencies were obtained within the harmonic approximation and are therefore expected to be systematically blue-shifted relative to the experimentally observed fundamental frequencies owing to the neglect of anharmonic effects. For comparison with experiment, representative empirical scaling factors were considered to account for the systematic overestimation of harmonic frequencies^[17]^. Table S1 summarizes the calculated harmonic frequencies, the scaled frequencies, and the corresponding experimental values for the different adsorbed species.

One point that needs to be addressed is that the calculated scaling factor (experimental value/calculation value) of isolated adsorbate is relatively smaller than the reported one. This is more prominent in the case of CH_2_OOH. However, this factor is quite sensitive to the adjacent environment. As given in the table, additional OH_b_ or water adsorption could induce a further blue shift of the computed vibrational frequency. The water environment is much more sophisticated than our simplified atomic models. Therefore, it’s reasonable to imply that the scaling factor will be closer to the reported one accounting for the realistic overlayed water adsorption in experiments.

Figure S9. Top (a) and side (b) views of the DFT-optimized structures of adsorbed methanol (CH₃OH), methoxy (CH₃O), and the proposed partially dehydrogenated intermediate CH₂OOH. Corresponding DFT-calculated C–H stretching vibrational frequencies are listed in Table S1.

Table S1. DFT-calculated C–H stretching vibrational frequencies of adsorbed CH₃OH, CH₃O, and CH₂OOH. The two values for Va_s_ come from the splitting of degenerate asymmetric frequencies due to additional OHb or water **(w)** adsorption.

| Adsorbed species | Vibrational mode | Calculated harmonic frequency (cm⁻¹) | Scaled frequency (0.95) | Experimental frequency/ Calculated frequency | Experimental frequency (cm⁻¹) |
| --- | --- | --- | --- | --- | --- |
| CH₃O | νₛ(CH₃) | 2995.1 | 2845.3 | 0.947 | 2839 |
| CH₃O | νₐₛ(CH₃) | 3086.1 | 2931.8 | 0.953 | 2940 |
| CH₃O+OH_b_ | νₛ(CH₃) | 2988.2 | 2838.8 | 0.949 | 2835 |
| CH₃O+OH_b_ | νₐₛ(CH₃) | 3078.5 | 2924.6 | 0.955 | 2940 |
| CH₃O+OH_b_+w | νₛ(CH₃) | 2984.4 | 2835.2 | 0.950 | 2835 |
| CH₃O+OH_b_+w | νₐₛ(CH₃) | 3065.4, 3079.1 | 2912.1,2925.1 | 0.959, 0.955 | 2940 |
| CH₃OH | νₛ(CH₃) | 3030.3 | 2878.8 | 0.941 | 2850 |
| CH₃OH | νₐₛ(CH₃) | 3143.0 | 2985.9 | 0.945 | 2970 |
| CH₃OH+OH_b_ | νₛ(CH₃) | 3027.6 | 2876.2 | 0.941 | 2850 |
| CH₃OH+OH_b_ | νₐₛ(CH₃) | 3123.5, 3136.9 | 2967.3,2980.1 | 0.951, 0.947 | 2970 |
| CH₂OOH | νₛ(CH₃) | 3044.0 | 2891.8 | 0.936-0.940 | 2850-2860 |
| CH₂OOH | νₐₛ(CH₃) | 3101.2 | 2946.0 |  | — |
| CH₂OOH+OH_b_+w | νₛ(CH₂) | 3031.5 | 2879.9 | 0.940-0.943 | 2850-2860 |
| CH₂OOH+OH_b_+w | νₐₛ(CH₂) | 3084.6 | 2930.4 | — | — |

Figure S10 presents all the calculated barriers of methanol’s first deprotonation for C1, C6 (a) and C2 (b) of Figure S8. The NEB calculation for C3 is not converged and is not present in this figure. In general, the water’s appearance near methanol is helpful for the first deprotonation as the barriers are lower (~ 0.1 eV) than that of vacuum condition, which is consistent with previous report. As shown in (b), the H-bonding’s direction will also influence the barrier as it’s 0.03 eV even lower than that of C1, where water’s position is same but with different orientations. It indicates that H-bonding plays an important role in the reduction of methanol decomposition.

**Figure 10:** The minimum energy path profiles of first methanol deprotonation for MeOH/water mix adsorption: (a) C1 and C6; (b) C2.

Figure S11 presents the possible adsorption structures of isolated methanol and isolated hydroxyl (above panel: terminal hydroxyl, O_t_H and down panel: bridging hydroxyl, O_br_H). Based on the relative energy, O_t_H is very unfavorable to form H-bond with methanol as it’s ~0.9 eV larger than those H-bonding with bridging O. However, O_br_H’s positions have little difference in total energy, which suggests that they could easily occupy various sites as its concentration increases.

**Figure S11:** Considered methanol and hydroxyl mix adsorption configurations and their relative energy.

Similarly, the barriers of methanol decomposition were also evaluated under the appearance of different hydroxyls as shown in Figure S12. Generally, O_br_H could promote methanol’s dissociation while O_t_H hinders the reaction. As given in (a), the dissociation barrier is 0.22 eV and 0.19 eV for C3 and C2 of Figure S11, respectively. They are both lower than that of vacuum environment. However, the barrier becomes 0.33 eV or even 0.69 eV for O_t_H co-adsorption. The distinct behavior may originate from the different charge status of the two cases. As is known, the introduction of O_br_H could induce excess electrons in the system while O_t_H could obtain excess holes. And the first deprotonation of methanol is more relevant to the excess electrons rather than holes. Therefore, the O_br_H could promote methanol’s dissociation.

**Figure S12:** The minimum energy path profiles of first methanol deprotonation for MeOH/hydroxyl mix adsorption. (a) and (b) correspond to O_br_H and O_t_H adsorption, respectively.

In addition, we also examined the influence of co-adsorption of both water and hydroxyl. In this case, two basic configurations of C1 and C6 from Figure S8 were adopted for adding hydroxyl as shown in Figure S12. Interestingly, the addition of hydroxyl on the surface could further reduce the deprotonation barrier almost in all cases except C2 based (C6), see Figure S14. Due to the sophisticated environment of multiple adsorbates, the barrier pathways are also complicated and sometimes show bumps along the reaction coordinate. However, the barrier reduction is quite prominent as shown in Figure S14a, where the barrier is of 0.03 eV and 0.07 eV for (C1) based substrate. Moreover, the reaction is barrierless for the indirect dissociation as given by Figure S14b. It means that the introduction of both water and hydroxyl could substantially lower the difficulty of methanol’s first deprotonation.

**Figure S13:** Considered methanol, water and hydroxyl mix adsorption configurations and their relative energy.

**Figure S14:** The minimum energy path profiles of first methanol deprotonation for MeOH/hydroxyl/water mix adsorption. (A) and (B) use the C1 and C6 water adsorption in Figure S6, respectively.

# References:

[1]	H. Onishi and Y. Iwasawa, "Reconstruction of TiO2(110) surface: STM study with atomic-scale resolution," *Surface Science* 313, no. 1-2 (1994): L783-L789. https://doi.org/10.1016/0039-6028(94)91146-0.

[2]	H. Onishi, K. Fukui and Y. Iwasawa, "Atomic-Scale Surface Structures of TiO2(110) Determined by Scanning Tunneling Microscopy: A New Surface-Limited Phase of Titanium Oxide," *Bulletin of the Chemical Society of Japan* 68, no. 9 (1995): 2447. https://doi.org/10.1246/bcsj.68.2447.

[3]	C. L. Pang, R. Lindsay and G. Thornton, "Structure of clean and adsorbate-covered single-crystal rutile TiO2 surfaces," *Chemical reviews* 113, no. 6 (2013): 3887. https://doi.org/10.1021/cr300409r.

[4]	K. Hermann, *LEEDpat download package*, Edmond, 2022.

[5]	W. Göpel, J. Anderson and D. Frankel et al., "Surface defects of TiO2(110): A combined XPS, XAES AND ELS study," *Surface Science* 139, no. 2-3 (1984): 333. https://doi.org/10.1016/0039-6028(84)90054-2.

[6]	H. S. Casalongue, S. Kaya and V. Viswanathan et al., "Direct observation of the oxygenated species during oxygen reduction on a platinum fuel cell cathode," *Nature Communications* 4, no. 1 (2013). https://doi.org/10.1038/ncomms3817.

[7]	N.-F. Chiu, C.-T. Kuo and C.-Y. Chen, "High-affinity carboxyl-graphene oxide-based SPR aptasensor for the detection of hCG protein in clinical serum samples," *International journal of nanomedicine* 14 (2019): 4833. https://doi.org/10.2147/IJN.S208292.

[8]	T. D. Kühne, M. Iannuzzi and M. Del Ben et al., "CP2K: An electronic structure and molecular dynamics software package - Quickstep: Efficient and accurate electronic structure calculations," *The Journal of chemical physics* 152, no. 19 (2020): 194103. https://doi.org/10.1063/5.0007045.

[9]	G. Lippert, J. Hutter and M. Parrinello, "The Gaussian and augmented-plane-wave density functional method for ab initio molecular dynamics simulations," *Theoretical Chemistry Accounts: Theory, Computation, and Modeling (Theoretica Chimica Acta)* 103, no. 2 (1999): 124. https://doi.org/10.1007/s002140050523.

[10]	S. Goedecker, M. Teter and J. Hutter, "Separable dual-space Gaussian pseudopotentials," *Physical review. B, Condensed matter* 54, no. 3 (1996): 1703. https://doi.org/10.1103/PhysRevB.54.1703.

[11]	J. P. Perdew, K. Burke and M. Ernzerhof, "Generalized Gradient Approximation Made Simple," *Physical review letters* 77, no. 18 (1996): 3865. https://doi.org/10.1103/PhysRevLett.77.3865.

[12]	J. P. Perdew, M. Ernzerhof and K. Burke, "Rationale for mixing exact exchange with density functional approximations," *The Journal of chemical physics* 105, no. 22 (1996): 9982. https://doi.org/10.1063/1.472933.

[13]	Z. Chai, G. Teobaldi, R. Si and L.-M. Liu, "Subspace Occupancy-Constraining Potentials for Modeling Polaron Formation," *The Journal of Physical Chemistry C* 125, no. 48 (2021): 26354. https://doi.org/10.1021/acs.jpcc.1c07709&ref=pdf.

[14]	S. Grimme, J. Antony, S. Ehrlich and H. Krieg, "A consistent and accurate ab initio parametrization of density functional dispersion correction (DFT-D) for the 94 elements H-Pu," *The Journal of chemical physics* 132, no. 15 (2010): 154104. https://doi.org/10.1063/1.3382344.

[15]	G. Henkelman, B. P. Uberuaga and H. Jónsson, "A climbing image nudged elastic band method for finding saddle points and minimum energy paths," *The Journal of chemical physics* 113, no. 22 (2000): 9901. https://doi.org/10.1063/1.1329672.

[16]	S. Liu, A.-A. Liu and B. Wen et al., "Coverage Dependence of Methanol Dissociation on TiO2(110)," *The journal of physical chemistry letters* 6, no. 16 (2015): 3327. https://doi.org/10.1021/acs.jpclett.5b01417.

[17]	J. P. Merrick, D. Moran and L. Radom, "An evaluation of harmonic vibrational frequency scale factors," *The journal of physical chemistry. A* 111, no. 45 (2007): 11683. https://doi.org/10.1021/jp073974n.
